# Elusive Differential of a Painful Unstable Total Knee Arthroplasty – Metal on Cement Disease

**DOI:** 10.7759/cureus.5495

**Published:** 2019-08-26

**Authors:** Vivek Jagadale

**Affiliations:** 1 Orthopedics, University of Arkansas for Medical Sciences, Little Rock, USA

**Keywords:** metallosis, cement disease, total knee arthroplasty, metal-on-polyethylene, mid-flexion instability

## Abstract

Particle disease from the release of metal, cement, ceramic, or polyethylene particles is a rare condition in total knee replacement. Wear, fracture, or corrosion of the components leads to foreign body reaction in the joint, followed by pain, swelling, limited motion, difficulty in walking, functional disability, and occasional systemic disease. This paper presents two cases of metal-on-cement disease as an etiology for the implant failure wherein the metal particles were shed off from the implant, along with cement and polyethylene particles, leading to a complex foreign body reaction in the failed total knee. We have coined this phenomenon as "cementallosis."

Initial symptoms and signs from implant failure in both patients were mild, intermittent mediolateral knee pain that was aggravated after walking but no pain at rest or at night. The pain was associated with a 'buckling' sensation, recurrent knee swelling, and gradually worsening knee range of motion. Clinical examination in both cases was suggestive of a well-healed anterior midline surgical scar, mild to moderate effusion, mild diffuse tenderness over the entire joint, limited functional range of motion, grade 1+ laxity throughout the range of motion, and intact distal neurovascular status. Routine blood and synovial fluid workup were inconclusive for identifying the exact etiology of the patient’s symptoms. Radiographs, computed tomography (CT) scans, as well as bone scans, were negative for prosthetic joint infection, component malalignment, polyethylene wear, maltracking, or loosening of the components but showed mild to moderate effusions, synovial hypertrophy, and mild scattered periprosthetic osteolysis. Intraoperatively, there was significant synovial hypertrophy with dark pigment deposition and apparent dissociation of the femoral and tibial components from the underlying cement mantle, although the cement was well-adhered to the underlying bone. Synovial fluid and multiple tissue specimens were suggestive of complex foreign body granulomas with metal, cement, and polyethylene particles. These patients had undergone surgery by the same surgery team in the community using the same implant.

These cases demonstrate the failure of a total knee arthroplasty implant at the metal-cement interface with features of adverse local tissue reactions that resemble a pseudotumor from the metal-on-metal disease in the knee joint. We have compared and contrasted the clinical presentations, laboratory, imaging, histopathological, and intraoperative study findings in these cases. Knowing what to look for will aid in early diagnosis, ordering necessary investigations, better surgery planning, reducing operative time, as well as improving outcomes and cost of care. The aim of this paper is to educate the audience about this new phenomenon as a cause of knee prosthesis failure produced by a complex pseudotumor-like foreign body reaction that involves metal, cement, and polyethylene particles.

## Introduction

Metal-on-cement disease is a rare condition in which corrosion and wear of the femoral, tibial, or metal-backed patella components occur due to broken cement-metal bonding in cemented primary or revision total knee replacement. Metal and cement debris further lead to third-body wear, and abrasive polyethylene wears, progressing to a slow and silent catastrophe. Existing literature lacks adequate information on the mechanisms and reasons for the failure of the total knee implant at the metal-cement interface. The implant failure commonly occurs from abrasion, fracture, fretting, fatigue, or adhesive wear [1]. This paper presents two cases who had unclear etiology for their implant failure and were later diagnosed with complex metal-on-cement disease.

## Case presentation

Case 1

A 78-year-old African-American obese male presented with progressively worsening mediolateral pain, buckling, difficulty in walking, and stiffness in the right extremity. His past surgical history included a right total knee arthroplasty (TKA) performed by a community orthopedic surgeon six years earlier (Smith & Nephew size 7 posterior stabilized femur component, size 6 tibial component with 9 mm polyethylene insert, 35 mm onlay patellar component, and low viscosity antibiotic-free bone cement) (Smith & Nephew, Inc., Cordova, TN, USA). He had presented multiple times to his primary surgeon with difficulty in walking but had a negative workup for the most common failure causes, such as prosthetic joint infection (PJI), instability, metal allergy, polyethylene wear, and aseptic loosening. His past medical history was positive for a body mass index (BMI) of 32.3, diabetes, hypertension, severe depression, and peptic ulcer disease. His clinical exam was suggestive of a well-healed anterior midline surgical incision, mild effusion, minimal generalized tenderness, non-tender on any ligament or tendon attachments around the joint, active knee range of motion from -10 degrees to 85 degrees flexion, grade 1+ mediolateral laxity throughout the range of motion, and intact distal neurovascular status.

Upon presentation to our clinic for a third opinion, his erythrocyte sedimentation rate (ESR) was within normal limits (13 mm/hr), C-reactive protein (CRP) was normal (6 mg/L), hemoglobin was low (12.1 gm %), serum leukocytes were normal (4,100/µL), knee joint aspirate revealed turbid hay-colored fluid with normal leukocytes (800/µL), neutrophils (NPs) (0%), red blood cells (70/µL), and a negative alpha-defensin assay. These findings were negative for prosthetic joint infection (PJI). The patient displayed no systemic signs of cobalt toxicity or infection.

**Figure 1:**
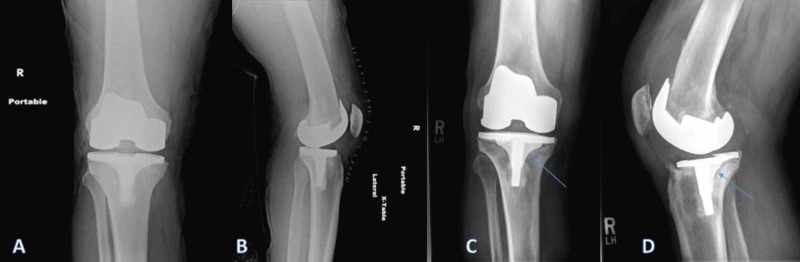
Right knee anteroposterior (AP) and lateral (LAT) radiographs taken immediately after the initial surgery (A, B) in comparison with those taken just prior to the revision surgery (C, D) showing minimal periprosthetic osteolytic areas underneath the tibial base plate with well-fixed cement-bone interface on the rest of the implant but no signs of metal-cement de-bonding

**Figure 2:**
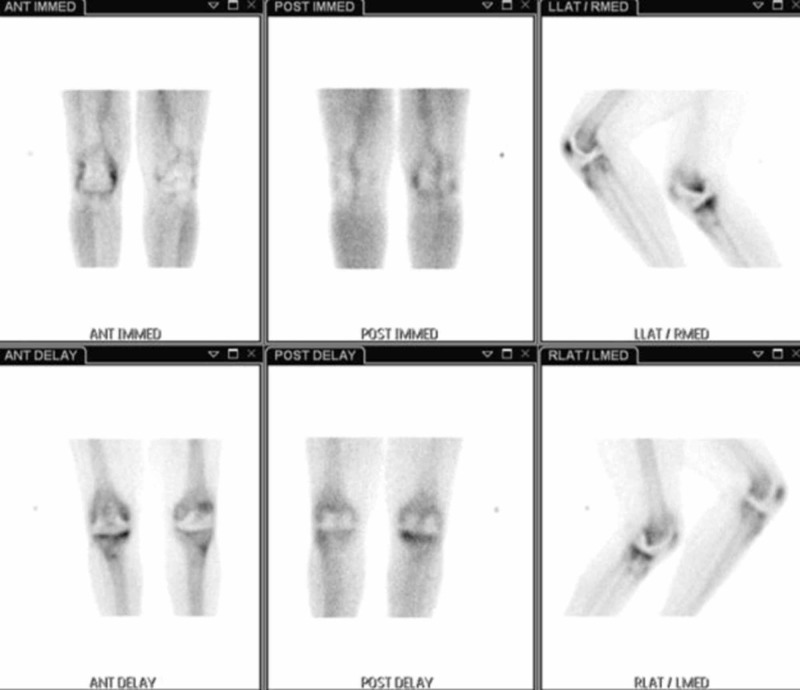
Three-phase bone scan shows increased radiotracer uptake in the periprosthetic region of the right knee on the venous phase of the flow, blood pool, and delayed series, which are consistent with periprosthetic osteolysis

**Figure 3:**
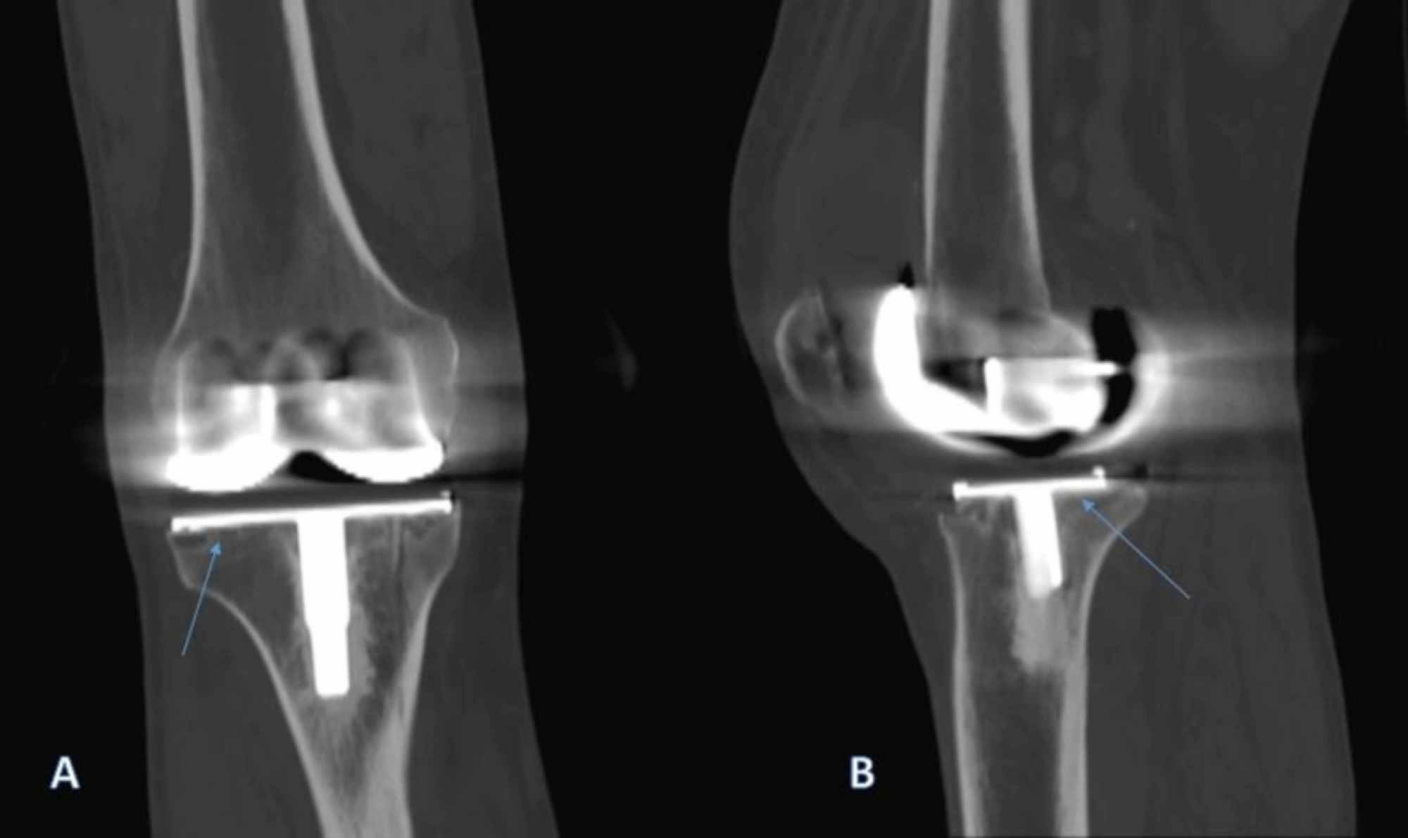
Computed tomography (CT) scan of the right knee Coronal (A) and sagittal (B) views show minimal periprosthetic osteolysis areas underneath the tibial base plate with no obvious loosening of the components

Routine radiographs of the knee joint (Figure 1), three-phase bone technetium scan (Figure 2), and computed tomography (CT) scans (Figure 3) were negative for any signs of component loosening, malalignment, component asymmetry, or maltracking and only showed moderate effusion.

**Figure 4:**
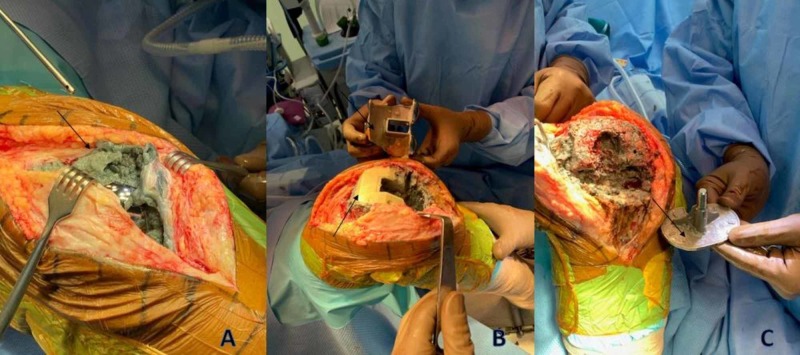
Intraoperative photographs of gross examination of tissue staining of pseudotumor from metal-on-cement disease

The patient had gradually worsening symptoms and wanted to have something done surgically to 'fix' his problem; however, the etiology of the failed implant remained unclear. We discussed our findings, available treatment options, their benefits, risks, alternatives, and complications, and the patient decided to pursue a single-stage vs. two-stage revision knee replacement surgery. Revision surgery was performed through the prior incision and arthrotomy; turbid hay-colored fluid gushed out of the joint in a projectile fashion. Severe metal debris and tissue staining with necrosis from metal-induced adverse local tissue reaction (ALTR) were noted throughout the knee (Figure 4A). The femoral, as well as the tibial components, could be removed easily without the need for any unique extraction device with good intact cement mantle, as well as bone stock (Figure 4B-C). The cement mantle was found to be firmly interlocked in the underlying bone, and there was no visible fracture of the implant, cement, or bone. The polyethylene patella component was well-fixed to the underlying bone and had to be removed with a thin saw blade. After thorough debridement, approximately 20% damage to the quadriceps mechanism was noted, and the tear was reinforced back to the native tendon with absorbable suture material. Extensive sharp debridement of the necrotic soft tissues was performed, and gross photographs were obtained. Many soft tissue and bone specimens from different locations inside the joint from underneath the femur and tibial components were obtained. The cement was removed meticulously with thin saw blades and flexible osteotomes, which helped in minimizing bone loss. Bone surfaces were prepared with saw and bone cutting guides, as well as curettes and bone ronguers. The joint cavity was thoroughly irrigated with 9 liters of 0.9% normal saline using the pulse lavage irrigator. Intraoperative frozen section specimens showed zero neutrophils/high-power fields (HPF) in six different fields of dense fibrotic tissue with scattered pigmentation and associated necrosis without evidence of acute inflammatory cells, abscesses, or vasculitis.

**Figure 5:**
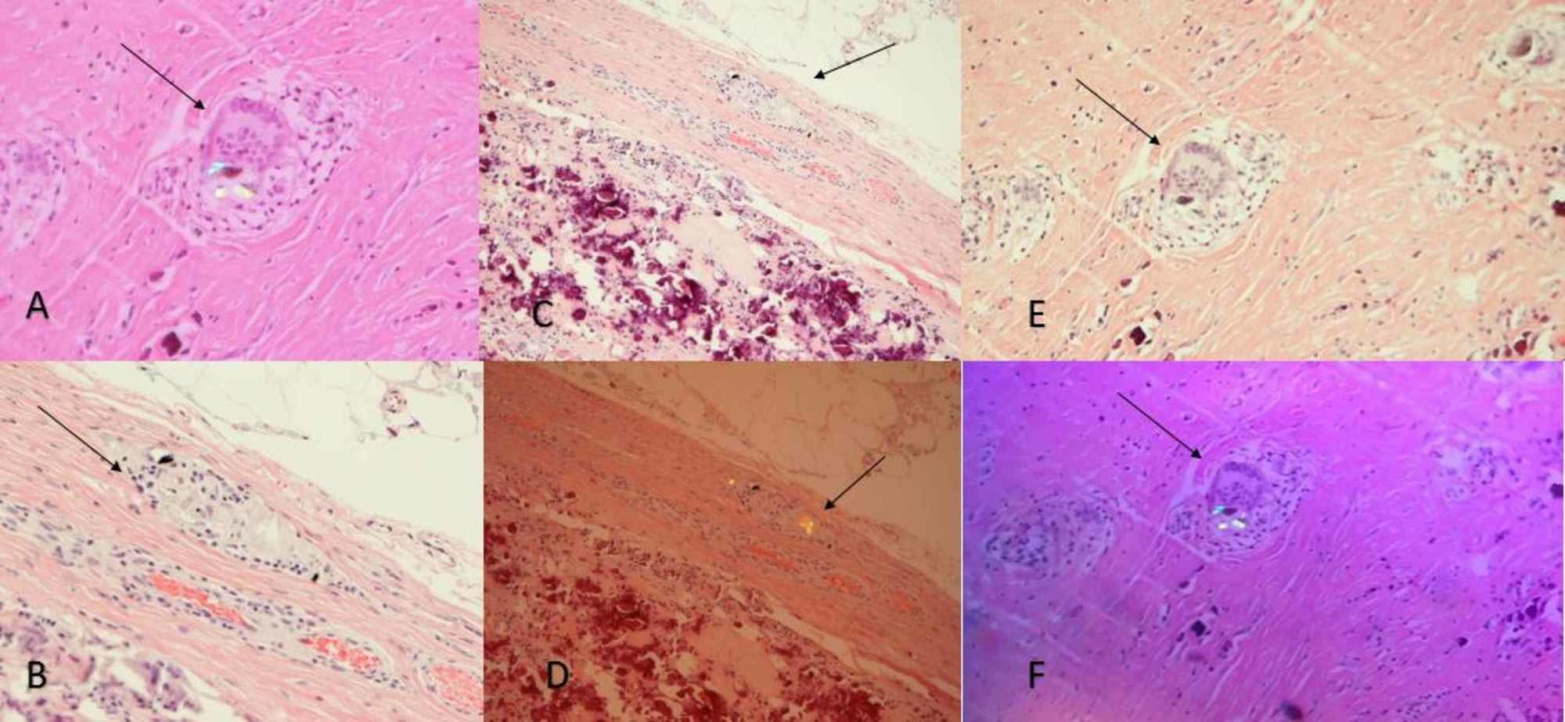
Histopathological slides with photomicrograph showing Type 1 histology of light to dark pigmented foreign body deposits under light microscopy (A, B, C) and under polarized light (D, E, F) in fibroconnective tissue with necrosis consistent with metal particle disease Hematoxylin and eosin stain, original magnification x100

After bone surface preparation, tourniquet inflation, implant sizing, and balancing flexion-extension gaps with trial implants, cemented short-stemmed components were implanted in the knee using low-viscosity tobramycin antibiotic-loaded bone cement. A standard polyethylene insert trial was placed until the cement hardened. The patella was left unresurfaced due to fear for patellar fracture. Adequate hemostasis was achieved before the beginning of the wound closure, a drain was not placed, and the wound was closed in layers in a regular fashion. Final cultures, as well as histopathological findings, were negative for infection but positive for adverse local tissue reaction resembling metallosis (Figure 5A-C) in combination with polyethylene particles (Figure 5D-F) and cement (Figure 5E-F) particles.

The differential diagnoses based on the clinical, laboratory, and radiological findings were low-grade chronic PJI, unbalanced TKA, implant malalignment, catastrophic polyethylene wear, periprosthetic osteolysis, or metal allergy. Metallosis-related pseudotumor was never in the differential diagnosis as there were no signs of metal-on-metal articulation or implant fracture that would cause metallosis. Postoperative radiographs showed an optimal alignment between the femoral and tibial components. The patient tolerated the procedure well and was subsequently admitted to the hospital for observation, pain control, routine antibiotics, and physical therapy.

An 81 mg twice daily aspirin tablet was started postoperatively as prophylaxis for deep vein thrombosis. The patient was evaluated, ambulated four hours after surgery by a physical therapist, and was on a walker weight-bearing as tolerated on the operated leg. The immediate postoperative range of motion was -5 to 95 degrees. He was discharged to the rehabilitation on postop Day 3 after tissue culture results came back negative and was instructed to continue exercises at home. On follow-up visit at two, six- and 12 weeks postop, the patient had successfully passed all phases of physical therapy and was doing well. His final cultures were negative. On the last follow-up at one year, the patient was ambulating four miles daily using his cane, his incision had healed nicely, and his knee stability, as well as the pain, had significantly improved. The patient was made aware of the findings, and his permission was obtained for submission of this case report.

Case 2

A 63-year-old obese Caucasian man (BMI 34.7), with a history of post-traumatic stress disorder, diabetes mellitus type 2, restrictive lung disease, tobacco abuse, and coronary artery disease, presented with a painful right primary total knee arthroplasty (Smith & Nephew Size 8 posterior stabilized femur component, size 7 tibial component with a 9 mm polyethylene insert and 38 mm onlay patellar component, with low viscosity antibiotic-free bone cement) seven years after his initial surgery. The clinical presentation was chronic moderate mediolateral knee pain, along with buckling, knee joint stiffness, and recurrent effusions, which started immediately after the initial knee surgery and had gradually gotten worse. On knee examination, he had similar findings as in Case 1 with an additional painful clicking sensation on moving his knee.

His ESR was within normal limits (9 mm/hr.), CRP was normal (5 mg/L), hemoglobin was normal (14.3 gm %), leukocytes were normal (4,100/µL), knee joint aspirate revealed turbid hay-colored fluid with slightly elevated leukocytes (1,150/µL), no NPs (0%), minimal red blood cells (750/µL), and a negative alpha-defensin assay. His imaging studies, including radiographs, CT scan, and bone scans, were suggestive of focal scattered areas of periprosthetic osteolysis on the tibial baseplate side but negative for all other components. With a strong suspicion for metallosis in the knee, we performed serum chromium (1.3 µg/L), a serum cobalt level (2.9 µg/L - n), and a metal artifact reduction sequence magnetic resonance imaging (MARS MRI) study for the knee before revision surgery was planned.

Upon incision through the previous anterior midline scar and medial parapatellar arthrotomy, dark hay-colored synovial fluid gushed out of the joint and significant pigmented synovial hypertrophy with dark metal debris with frank de-bonding of the femoral and tibial components from the underlying cement mantle were noted. The cement mantle appeared to be well-adherent to the underlying bone. Extensive pseudotumor removal and joint debridement were achieved. The infection was ruled out intraoperatively by frozen section evaluation, and after satisfactory joint preparation, cemented short-stemmed tibial and femoral components were implanted. Final synovial, bone, and soft tissue cultures, as well as histopathological photomicrographs of the tissue slides, were negative for infection but positive for metal, cement, and polyethylene particle-related granulomas, as seen in Case 1.

## Discussion

Metal-cement interface failure in cemented total knee arthroplasty with pseudotumor formation in the knee is not a common mechanism of knee implant failure and has not been reported in the literature. The de-bonding between the metal component and underlying cement could be triggered by a variety of reasons, including weak quality cement, poor cement mixing techniques, inadequate immobilization of the knee joint while the cement is curing, or inadequate cementing non-low viscosity cement use [1-2]. This interface failure can cause gradual loosening and dissociation of components with neighboring ALTR that could mimic PJI, inflammatory synovitis, or tumor formation occasionally, along with systemic signs of metallosis, but they rarely coexist [3]. In our observation, the clinical presentation, laboratory studies, radiological impressions, histopathological findings, and intraoperative findings in both patients were markedly similar. After identifying the etiology of the implant failure, both patients were managed successfully by single-stage revision total knee replacement with excellent outcomes.

Although metallosis and polyethylene wear disease in the hip have been adequately reported in the literature, there is not much information on a similar phenomenon in the knee and the literature lacks evidence on the typical clinical presentations, investigations, and approaches to the diagnosis or management options [3]. Also, there is limited information on outcomes of particle disease involving cement, ceramic disease, or even their combination that could be causing a myriad of pathological changes in the adjacent bone and soft tissues [4]. Literature evidence suggests that metallosis in a knee is possible from breakage of polyethylene, metal fracture, insert displacement, failed metal-backed patella implant, cementless total knee arthroplasty designs with manufacturing issues, modular hinged knee designs, and saw blade debris-related foreign body reaction [3-5]. Ongoing abrasion, corrosion, and wear at the metal-cement junction can cause shedding of metal or cement particles into the joint from abrasive wear, leading to adhesive third-body wear of the polyethylene insert and subsequent signs of joint failure [2, 5]. The local adverse reaction not only involves the destruction of the synovium, capsule, ligaments, tendons, and bone but also involves implant instability, loosening, joint destruction, and systemic signs of metal poisoning [5]. On gross examination of the inside of the knee, there is visible dark pigmentation and hypertrophy of the synovium with a shiny, smooth, intact cement mantle on the rear side of the loose implant and strong cement adherence to most of the underlying bone [6]. These metal, cement, and polyethylene particles are cytotoxic and, when phagocytized, lead to soft tissue and occasional bone necrosis [3]. The sequelae of this phenomenon are defined as an aseptic, lymphocyte-dominant, vasculitis-associated lesion (ALVAL) or adverse local tissue reaction (ALTR), also known as a pseudotumor, in isolated metal particle disease. This appears radiologically as a walled-off fluid collection and clinically manifests as joint distention and vague pain [3]. Patients are required to be monitored for signs of systemic cobalt or chromium toxicity in the form of worsening neurological symptoms, such as drowsiness, depression, psychosis, and dementia [4]. We do not know clearly if cement or polyethylene wear could cause any systemic signs and symptoms, and if so, what we should be looking for in the blood.

Modern total knee arthroplasty is a very successful operation with 96% overall patient satisfaction [7]. However, implant designs, surfaces and combinations, component assembly, joint preparation, implant sizing, and placement techniques in the body with regards to size, orientation, symmetry, balance, alignment, combinations, and offsets influence the longevity of the implant. Modular knee prostheses with a variable level of constrains, hinges, cones, and stems allow more flexibility while doing complex primaries and revisions, but they require more meticulous planning, expertise, and execution. Due to these and numerous other reasons, uncemented total knee replacement is also slowly and steadily gaining popularity; however, the indications are still limited. Monobloc all-polyethylene tibial components, less constrained polyethylene tibial inserts, and rotating platform tibia designs are occasionally used for specific indications, but the indications are limited and the goal is to reduce particle disease-related component failure [8].

Patients with metal-on-cement disease present primarily with chronic diffuse knee pain, buckling, and difficulty in walking; thus, a broad subset of etiologies must be considered [3, 7]. The differential diagnoses are a chronic low-grade PJI, instability, aseptic loosening of components from polyethylene wear, implant misalignment, inflammatory joint disease, metal allergy, chronic pain syndrome from a chronic connective tissue disorder, local neoplastic process, or insufficiency fractures [2, 9]. MARS MRI or metal ion levels are not done routinely in knees due to the nature of metal on polyethylene articulation but are important as it will help diagnose hidden etiologies for implant failure, as seen here. A treatment algorithm indicating an approach to a patient with painful instability is available but may need an update based on these findings [10-11]. Extensive debridement to remove the dead necrotic tissues is necessary. Often, it is possible that all three particles are scattered throughout the joint in variable concentrations causing all sorts of complex foreign body reactions in the soft tissues and bones. We have coined this unique clinical, diagnostic, intraoperative, and histopathological presentation from complex particle disease as ‘cementallosis.’

PJI can be a devastating complication of knee arthroplasty, and at times, the diagnosis can be very elusive [12-13]. The major and minor diagnostic criteria set by the Musculoskeletal Infection Society (MSIS) have been beneficial in most of the situations, but a diagnosis could get tricky in simultaneous particle disease, leading to highly vascularized pseudotumors, hemarthrosis, bloody aspirate, or low-grade infections from fungus or bacteria that could have a significant impact on surgical planning, complications, morbidity, mortality, and cost of care [14]. Alpha-defensins are antimicrobial peptides released by NPs in response to pathogens, and they have been used more often than ever to identify such infections [15-17]. They can be measured in synovial fluid and are released from NPs in response to a variety of conditions, including infections, foreign body reactions, and interstitial lung diseases. Alpha-defensin use as a PJI diagnostic marker was introduced first by Deirmengian et al. in 2014 [12]. Aspirates from prosthetic joints with metallosis demonstrate approximately a 30% false-positive alpha-defensin rate, so it must be kept in mind while ruling out PJI in knees [15]. A bloody tap can skew the synovial fluid NP, as well as the leukocyte count, increasing the possibility of a false-positive interpretation of PJI [17]. Often, the tissue cultures return negative for growth of microorganisms, and if this is the only criteria set used, then patients could be getting 'unnecessary' extensive two-stage surgeries.

On histopathological examination proposed by Moraweitz et al., the periprosthetic membrane can be classified into four different etiological types [6]. Type I histology is characterized by the presence of foreign-body particles, macrophages, and multinucleated giant cells, typical for wear-particle disease; Type II histology is defined by the presence of NPs and a very few foreign body particles, typical for an infectious etiology; Type III is a combination of Types I and II, while Type IV histology is indeterminate. Algeorge et al. noted a good correlation between microbiology and histology concerning the diagnosis of infection [18]. All cases of microbiologically-confirmed PJI had a heavy polymorph infiltrate with > 5 NPs per HPF in periprosthetic tissues based on this study. They noted occasional NPs in some cases of aseptic failure, mostly < 1 NP per HPF on average, but in one case, up to 4 NPs per HPF were noted. Often, osteolysis is masked by obesity, pseudotumor formation on x-rays, as well as CT scans [3]. Therefore, if telltale signs of metallosis, such as abnormal skin hyperpigmentation, radio-opaque effusion, and abnormal color and density of the joint fluid, are present, then adequate surgery planning by ordering additional investigations is essential. The bone scan is often false-positive for PJI so metallosis must be suspected for better surgical planning [19].

In the first patient, 'cementallosis' was never suspected so surgery planning was inadequate, in our opinion. During the second case with a similar clinical, laboratory, and imaging presentation, we decided to perform plasma cobalt and plasma chromium tests, as well as to obtain a MARS MRI. The metal-cement disease in both cases was seen primarily affecting the femoral component. MARS MRI and plasma cobalt/chromium levels are typically not checked due to low suspicion for metallosis due to the nature of the standard knee prosthesis articulation. CT scan, bone scan, and Indium scan could all be occasionally inconclusive for identifying the etiology of implant failure. 

## Conclusions

Metal-on-cement disease in the cemented total knee arthroplasty demonstrates the striking features of complex adverse local tissue reaction from metal, cement, and polyethylene particles. This occurs primarily due to abrasive wear caused by metal-cement de-bonding and grinding type wear from the combined shear with compressive forces at the artificial knee joint during the stance phase of walking. The diagnostic dilemma could be easily resolved by knowing the etiologies of implant failure that could be responsible for painful, unstable, swollen, stiff knee arthroplasty. The aim of this paper is to educate the audience with possible symptoms, signs, approach, and diagnostics that can assist in identifying the often elusive etiology of painful, unstable knee arthroplasty that could be particle disease from metal-on-cement interface failure. This information will not only aid in early diagnosis and management but also help in improved patient counseling, improved outcomes, reduced morbidity of multiple complex surgeries, and reduced cost of care, especially when the etiology of implant failure is an enigma.
